# Impact of Internet usage time on mental health in adolescents: Using the 14^th^ Korea Youth Risk Behavior Web-Based Survey 2018

**DOI:** 10.1371/journal.pone.0264948

**Published:** 2022-03-23

**Authors:** Yeunhee Kwak, Hyejin Kim, Jung-Won Ahn

**Affiliations:** Red Cross College of Nursing, Chung-Ang University, Seoul, Korea; Universitat de Valencia, SPAIN

## Abstract

Dependency on the Internet in daily life is increasing, and the negative consequences this dependence may have on mental health are not sufficiently understood. The aim of this study was to investigate the relationship between Korean adolescents’ Internet usage time and their mental health. This cross-sectional study included 29,811 high school students ages 16–18 from the 2018 Korea Youth Risk Behavior Web-Based Survey. Participants’ mean Internet usage time was 193.4±1.6 min/day. Internet usage time was associated with sex, grade level, type of school, living arrangement, economic status, academic achievement, and experience of school violence. With regard to mental health, subjective health status, stress, feelings of sadness, and suicidal ideation were also related to Internet usage time. The group with more than average Internet usage had poorer subjective health, higher level of stress, and had feelings of sadness and suicidal ideation compared to the group with less than average Internet usage. To effectively manage Internet usage time, interventions to lower Internet usage and leisure programs that could replace Internet usage need to be developed.

## Introduction

With innovative advances in the information technology industry, the Internet penetration rate in most developed countries exceeds 90%, and the number of Internet users is consistently rising [1]. With the recent coronavirus pandemic, Internet use among adolescents has further increased. Adolescents are familiar with computers and they also utilize the Internet for various purposes. As the Internet is used as means for a multitude of activities, including education, communication, entertainment, and online communities, it is becoming more intricately woven into our daily lives; in particular, its influence on the lives of adolescents is not negligible [2, 3]. While the Internet has a variety of favorable functions, such as sharing of information, communication, and stress relief, excessive Internet usage can create issues such as dependence and interference with daily living, thereby posing a significant public health problem [3, 4].

Adolescence is a transitional period in which individuals are required to adjust to drastic physical, mental, and social changes, and adolescents also undergo various crises related to academic stress, peer relations, and school adaptation [5, 6]. Internet usage in adolescence has been shown to be associated with emotional and physical problems [7, 8]. Excessive Internet usage causes problems in daily living and may have an adverse impact on mental health [5, 8]. As adolescents are inquisitive, lack self-control, and have yet to establish a self-identity, they are vulnerable to becoming dependent and addicted to Internet use. Research has found excessive Internet usage in adolescence to be associated with psychosocial problems, such as failure to distinguish between the real world and the virtual world, maladaptation in life, poor academic progress, and avoidance of interpersonal relationships, as well as excessive fatigue and reduced sleep duration [2, 9, 10]. One of the most important components of defining addictive Internet usage is nonessential excessive Internet usage time; as problematic Internet use (PIU) increases with Internet usage time [15, 16], there is a need to investigate the status of adolescents’ Internet usage and those factors that are associated with excessive Internet usage.

Adolescents today complain of a high level of stress as they undergo rapid physical and psychological development. In addition, the education-centered environment that surrounds adolescents can cause negative emotions such as depression and anxiety [10, 11], and such negative emotions may lead to risk-taking behaviors [4, 7]. In an attempt to avoid stressful situations, some adolescents focus on the Internet, which provides a virtual world, that may lead to Internet dependence [10, 11]. Adolescents’ psychological traits have been found to predict excessive Internet usage, and their Internet usage has surfaced as an important societal issue [12]. Previous studies have ambiguously classified Internet usage time into low, moderate, and high and have failed to present consistent criteria for Internet usage time [8, 9]. Moreover, despite the rapid increase in the rate of educational Internet usage, past studies simply surveyed total Internet usage without distinguishing the purpose of Internet use into learning and non-learning purposes, which has made interpretation of their findings difficult. In the present study, we examined the association between non-academic Internet usage time and mental health among adolescents, specifically high school students, using the raw data from the 2018 Korea Youth Risk Behavior Web-Based Survey (KYRBWS-14). The findings of the study would have implications for interventions and programs to promote adolescents’ mental health.

The specific objectives of the study were as follows: (1) examine participants’ general characteristics, Internet usage time, and mental health; (2) examine differences in general characteristics and mental health according to Internet usage time; and (3) examine the association between Internet usage time and mental health.

## Materials and methods

### Study design and participants

A cross-sectional design was used to investigate the association between Internet usage time and mental health (subjective health status, stress, feelings of sadness, and suicidal ideation) among Korean adolescents. Although physical health is not a mental health variable, we included subjective health status as a health indicator that has importance for mental health.

The KYRBWS is an anonymous self-reported online survey of youth in the first year of middle school to the final grades of high school extracted via a complex sample design such as stratification, clustering, and multistage sampling methods. The raw data from the KYRBWS-14 conducted by the Korea Disease Control and Prevention Agency (KDCA) were analyzed. The KYRBWS is a government approved statistical survey (Statistics Korea, approval No. 11758) and was approved by the IRB of the KDCA (2014–06EXP–02–P–A).

The KYRBWS-14 was conducted from June 1–31, 2018. We obtained the raw data from the KYRBWS website (https://www.kdca.go.kr/yhs/) and received approval for its use from KDCA. Of 62,823 students in 400 middle schools and 400 high schools, 60,040 (95.6%) students participated in the survey. In this study, data from 29,811 high school students (15 135 boys, 14,676 girls) ages 16 to 18 were analyzed after excluding participants with missing values.

### Measurement

#### Internet usage time

Participants were asked, “In the last 30 days, on average, how many hours per day did you use the Internet for non-academic purposes?” The daily average duration of Internet usage including weekdays and weekends were converted to minutes. With reference to the mean Internet usage time in our participants (193.4±1.6 minutes/day), the participants were divided into the below average usage group and the above average usage group.

#### Mental health

Mental health was evaluated using subjective health status, stress, feelings of sadness, and suicidal ideation. Regarding subjective health status, students were asked “how do you rate your health?” Responses of “very good,” “good,” and “moderate” were considered “good” health, and “poor” and “very poor” were considered “poor” health. Stress was measured by asking students how much stress they normally have, and “very high” and “high” were classified as “yes,” and “a little” and “almost none” were classified as “no.” “Feelings of sadness” was measured using a yes-or-no question about whether they felt sad or hopeless to the point of being unable to carry on with their normal daily living for two consecutive weeks in the past 12 months. Suicidal ideation was measured using a yes-or-no question about whether they had seriously thought about committing suicide in the past 12 months.

#### General characteristics

Sex, grade level, type of school (high school, vocational high school), living arrangement (with family, with relative, live alone, dormitory, children’s home), economic status, academic achievement (high, moderate, low), and experience of violence (yes, no) were surveyed.

### Data analysis

Statistical analysis were performed using the SAS software 9.4 (SAS Institute Inc., Cary, NC, USA) in accordance with the KDCA guidelines on sampling weights and nationally representative estimates. Above average Internet usage was defined by first computing the mean Internet usage time among the participants and dividing them into above average and below average groups. Continuous variables were presented in means (*SE*), and categorical variables were presented in percentages (*SE*). General characteristics according to Internet usage were analyzed using chi-square tests. The association between Internet usage time and mental health was analyzed with logistic regression. Odds ratios (OR) and confidence intervals were estimated in model 2 after adjusting for general characteristics. Above average Internet usage was determined using a cutoff. Reference value 1 is the group that uses Internet greater than the mean duration of 193.4±1.6 min/day.

## Results

Table 1 shows the demographic characteristics of the participants according to Internet usage time. With regard to the above average Internet usage group, 3,508 (12.3%) were male users and 4,138 (13.5%) were female users. Internet usage time differed according to sex, grade level, type of school, living arrangement, economic status, academic achievement, and experience of violence. Internet usage was highest among students in lower grade levels (*p* < .001), female students (*p* < .001), vocational high school students (*p* < .001), students living in a children’s home (*p =* .006), students of low economic status (*p* < .001), low academic achievement students (*p* < .001), and those students who had experienced violence.

**Table 1:** Demographic characteristics by daily Internet usage (N = 29,811).

Variable	Classification	Daily Internet usage (min)	n (%)	Below average user	Above average user	*t* or *F* (*p*)
		Mean ± *SE*^*a*^		n (%)	n (%)	
Sex	Male	191.8 ± 2.1	15135(50.8)	11627(39.0)	3508(11.8)	45.61 (< .001)
	Female	194.8 ± 2.1	14676(49.2)	10538(35.3)	4138(13.9)	
Grade level	1^st^	197.5 ± 2.3	9260(31.1)	6795(22.8)	2465(8.3)	6.18 (.002)
	2^nd^	195.7 ± 2.1	10039(33.6)	7404(24.8)	2635(8.8)	
	3^rd^	188.3 ± 2.2	10512(35.3)	7966(26.7)	2546(8.6)	
School type	High school	185.4 ± 1.7	23882(80.1)	18027(60.5)	5855(19.6)	41.79 (< .001)
	Vocational high school	237.8 ± 4.1	5498(18.4)	3793(12.7)	1705(5.7)	
Living arrangement	With family	193.7 ± 1.6	26984(90.5)	19980(67.0)	7004(23.5)	3.55 (.006)
	With relatives	205.3 ± 11.7	222(0.8)	170(0.6)	52(0.2)	
	Alone	232.1 ± 13.3	280(0.9)	210(0.7)	70(0.2)	
	Dormitory	175.3 ± 6.0	2187(7.3)	1706(5.7)	481(1.6)	
	Children’s home	349.4 ± 26.5	138(0.5)	99(0.3)	39(0.2)	
Economic status	High	173.7 ± 2.2	10503(35.2)	8314(27.9)	2189(7.3)	102.32 (< .001)
	Moderate	194.9 ± 1.7	14354(48.2)	10529(35.3)	3825(12.9)	
	Low	253.5 ± 3.9	4954(16.6)	3322(11.1)	1632(5.5)	
Academic achievement	High	173.7 ± 2.2	10125(34.0)	8009(26.9)	2116(7.1)	76.73 (< .001)
	Moderate	194.9 ± 1.7	16310(54.7)	12072(40.5)	4238(14.2)	
	Low	253.5 ± 3.9	3376(11.3)	2259(7.6)	1117(3.7)	
Violence experience	Yes	205.9 ± 11.9	553(1.9)	473(1.6)	80(0.3)	24.53 (< .001)
	No	193.2 ± 1.6	29258(98.1)	21692(72.7)	7566(25.4)	
Subjective health status	Good	184.2 ± 1.6	20154(67.6)	15698(52.7)	4456(14.9)	244.42 (< .001)
	Poor	210.7 ± 2.1	9657(32.4)	6642(22.3)	3015(10.1)	
Perceived stress	Yes	194.3 ± 1.6	4646(15.6)	3637(12.2)	1009(3.4)	36.53 (< .001)
	No	187.9 ± 2.5	25165(84.4)	18528(62.2)	6637(22.2)	
Feelings of sadness	Yes	201.9 ± 2.2	8549(28.7)	6145(20.6)	2404(8.1)	25.86 (< .001)
	No	189.8 ± 1.7	21262(71.3)	16020(53.7)	5242(17.6)	
Suicidal ideation	Yes	216.3 ± 3.6	3834(12.9)	2603(8.8)	1231(4.1)	62.91 (< .001)
	No	189.8 ± 1.6	25977(87.1)	19562(65.6)	6415(21.5)	

^a^SE = standard error of mean.

**Fig 1:**
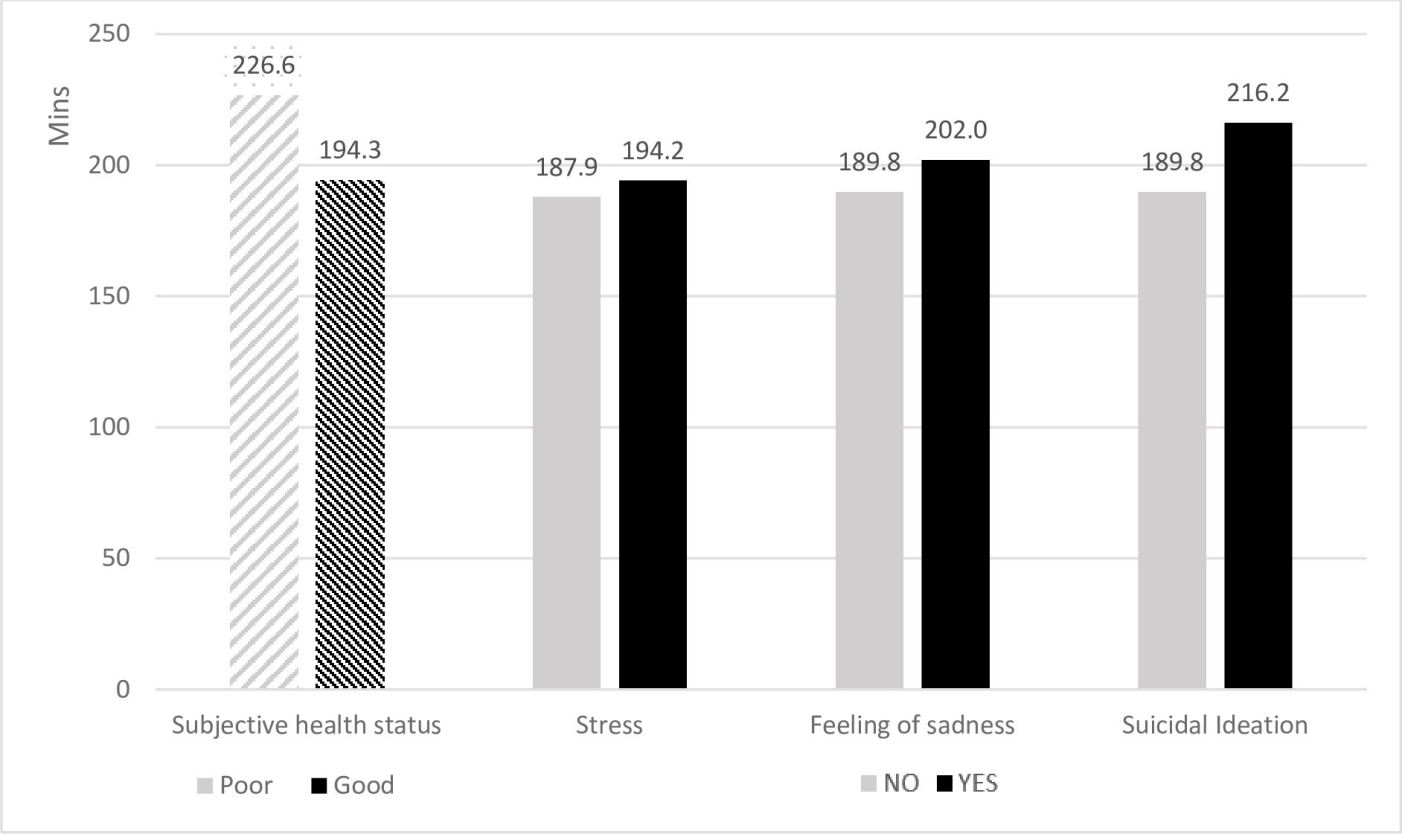
Association between daily Internet usage time, subjective health status, stress, feeling of sadness, and suicidal ideation among adolescents.

Internet usage time differed according to subjective health status, stress, feelings of sadness, and suicidal ideation. Internet usage time was longer among those who reported having poor health (*p* < .001), stress (*p* < .001), feelings of sadness (*p* < .001), and/or suicidal ideation (*p* < .001). Fig 1 shows the differences in Internet usage time according to the mental health variables.

Table 2 shows the relationships among Internet usage time, subjective health status, stress, feelings of sadness, and suicidal ideation. Model 2 examined the relationship between Internet usage time and mental health after adjusting for grade level, sex, academic achievement, living arrangement, economic status, and type of school. In model 2, the group with less than average Internet usage time had an OR of 0.67 (0.63–0.71) for subjective health status, 0.83 (0.76–0.90) for stress, 0.90 (0.84–0.96) for feelings of sadness, and 0.75 (0.69–0.82) for suicidal ideation. Therefore, even after adjusting for the confounding variables, the group with more than average Internet usage had poorer subjective health, greater stress, and had feelings of sadness and suicidal ideation compared to the group with less than average Internet usage.

**Table 2:** Logistic regression for daily Internet usage on mental health.

		Subjective health status	Stress	Feeling of sadness	Suicidal ideation
Model 1^b^ Internet usage time	Above average usage	1	1	1	1
	Below average usage	0.63 (0.59–0.67)	0.81 (0.75–0.88)	0.88 (0.82–0.93)	0.74 (0.68–0.80)
	*p*-value^a^	< .001	< .001	< .001	< .001
Model 2^c^ Internet usage time	Above average usage	1	1	1	1
	Below average usage	0.67 (0.63–0.71)	0.83 (0.76–0.90)	0.90 (0.84–0.96)	0.75 (0.69–0.82)
	*p*-value*	< .001	< .001	< .001	< .001

^*a*^*p*-value of Likelihood Ratio test.

^b^Model 1: Adjusted for grade level and sex.

^c^Model 2: Adjusted for grade level, sex, academic achievement, living arrangement, economic status, and type of school.

## Discussion

This study contributes to broadening our understanding of adolescent mental health by comparing adolescents’ mental health according to Internet usage time. It sheds light on the need for further attention and interventions to promote adolescents’ mental health according to the extent to which the Internet is used for non-academic purposes.

In our study, the daily average non-academic Internet usage time was 193.4±1.6 min/day among high school students, and the average duration was longer at the lower grade levels. In a report providing statistics on Korean young people in 2018, adolescents used the Internet for 152 min/day and young adults in their 20s used the Internet for 207 min/day [13]. High school students use the Internet more than adolescents of other stages and ages do, and it is likely that the reduction of Internet usage for non-academic purposes in the last year of high school would be related to preparing for college admission.

In other countries, it has been found that adolescents in higher grade levels tend to use the Internet more, and one common aspect between adolescents from other countries and Korean adolescents is that they use instant messenger and social media the most [14]. Adolescents usually access the Internet using their cellphones, and the increased smartphone penetration rate in recent years has markedly contributed to an increase in Internet usage time.^1^ Adolescents mostly use the Internet for enjoying and passing time, and problematic Internet usage (PIU) is higher among Internet users who use the Internet for this purpose [15]. One of the most important components of defining addictive Internet usage is unessential excessive Internet usage time, and as PIU increases with Internet usage time [15, 16], excessive Internet usage should be managed properly.

Our results showed significant differences in sex, grade level, type of school, living arrangement, economic status, academic achievement, and experience of violence between the below average usage group and above average usage group. Adolescence is a period marked by the development of individual tendencies and the greatest environmental and parental influence on psychosocial development [17]. Our results showed that adolescents who lived with their family or lived in a dorm used the Internet substantially less than their counterparts who lived alone or lived in a children’s facility. This is consistent with previous results showing that a lack of parental, managerial support and control was associated with increased Internet usage time [6]. In this study, Internet usage time was markedly higher in the low economic status group compared to the high economic status group. Special attention may be needed for students with poor economic conditions and lack of supervisory care from surrounding people, which include parents, teachers, and friends. School and government may need to provide appropriate support to provide a stable living environment as necessary. Previous studies found that the high PIU group had lower academic achievement [14], and that there was a positive correlation between PIU level, general procrastination and academic procrastination [9, 15]. Our results showed that students with poor academic achievement had greater Internet usage time, which calls for Internet usage time management according to the level of academic achievement. Schools may offer after school or holiday supplementary study programs for students in lower academic levels to maintain efforts and interests in learning.

Excessive Internet usage among adolescents was reportedly related with diminished overall physical and psychological functioning, which may lead to a deterioration of social skills in real life, and as a result, these adolescents experience social isolation [4, 18]. Furthermore, it may worsen learning habits, thereby decreasing academic performance while increasing impulsiveness and aggression [19]. While research suggests that Internet usage can have a negative effect on adolescents’ academic performance, social relationships, and emotional wellbeing, the Internet is also an important source of information for adolescents [20]; therefore healthy Internet usage should be recommended rather than limiting Internet usage time coercively.

The results showed that compared to the below average usage group, the above average group had poorer perceived health, high stress and feeling of sadness, and a higher risk for suicidal ideation. Adolescents have poor health awareness and make little effort to maintain and promote their health, which can negatively affect their physical and mental health [21]. Our findings indicate that those who perceived they had poor health tended to use the Internet more. Therefore, it is necessary to help adolescents build confidence in their health by promoting physical activities [12]. Several studies have confirmed the association between excessive Internet usage and anxiety, depression, sleep quality, and physical activity [9, 10, 15]. Further, poor mental health and low physical activity can negatively affect subjective health status [5]. A study on European adolescents reported an association among obesity, excessive Internet usage, addictive behaviors, and low academic performance [8], highlighting the need for public health policies to promote physical activity and reduce Internet dependence from early adolescence.

In a study by Günlü and Ceyhan, adolescents pointed to obtaining information, communication, and fun as the positive influences of the Internet, and reported a waste of time, health problems, and cause of addiction as the negative influences of the Internet [15]. However, among those with PIU, acquiring information was ranked low in the list of purposes of Internet usage. With increasing time spent on the Internet, individuals may have difficulty with developing healthy interpersonal relationships and have less time to engage in social activities, contributing to depression [19]. There is an important association between PIU, adolescents’ loneliness, and social anxiety, as anxiety and depression interact with PIU [22]. When perceived stress increases, individuals may attempt to relieve the stress by overusing their smartphone, but as such relief is only transient, a feeling of sadness increases from the unresolved stress [23, 24]. Adolescents further focus on the Internet, which provides them with a virtual reality to avoid the stressful situation, but excessive Internet usage actually further elevates stress and depression [10, 25]. Ultimately, excessive Internet usage causes problems in daily living, deteriorates mental health, and has a detrimental impact on physical health and adolescent growth [10, 17]. Thus, various programs need to be developed that can help identify the main stressors of adolescence, such as career decisions, college entrance exams, peer conflict, help with counseling, and ongoing support.

Although not everyone who has suicidal thoughts plans or attempts suicide, those who do are at an increased risk of dying from suicide; continuous suicidal ideation is a factor in impulsive suicide attempts [26]. This study showed adolescents who reported experiencing suicidal ideation spent more time using the Internet. The Internet provides easy access to information about suicide [27], and people interested in suicide can actively search for information on the web. It has been reported that 16% of 339 suicide-related websites searched on Google provide information about suicide and suicide-related resources, and 14% of these websites provide specific methods and guidelines for suicide [28]. In fact, 73.4% of Korean people with a history of mental illness or suicidal ideation have been found to search for information on the Internet before seeking professional help [29]. Research showed that feelings of sadness and stress, the most important predictors of suicidal ideation, mediated the relationship between adolescents’ Internet usage and suicidal ideation [26]. Additionally, Cheng et al.’s meta-analysis indicated that the rate of suicidal ideation, planning, and attempts was significantly higher among those with Internet addiction, and the severity of suicidal ideation was also higher in this group [30]. It is necessary to explore further causes and influencing factors for adolescents’ suicidal ideation and to offer ways and resources to prevent them in schools and communities. Furthermore, it is crucial to monitor and impose sanctions on harmful websites that may provide harmful information or misinformation relative to suicide.

To prevent the adverse impact of excessive Internet usage among adolescents, active support and interventions are needed to reduce stress and feeling of sadness. It is necessary to provide mental health services that can continuously monitor and manage adolescents’ psychological health status such as stress, depression; maladaptation at school and poor peer relationships; and excessive Internet usage within the school and community. Further, it is also necessary to provide education to parents regarding Internet safety and parental roles, and counseling for school teachers. Policy measures should be prepared so that community mental health nurses and school health teachers collaborate to improve mental health management within schools. In addition, the educational environment needs to be ameliorated such that students can replace Internet use with other social and physical activities, as opposed to piecemeal policies that simply shorten Internet usage. Further, self-management programs that promote healthy use of the Internet, and interpersonal relationship programs that help adolescents maintain good relationships are also needed. Internet filter software needs to be continuously managed and upgraded at the national level to block harmful information or websites considering the user’s age group.

### Strengths and limitations

This study is meaningful in that data were collected on a large scale, from among national data, and analyzed to identify associated factors based on the average amount of Internet usage time that is being directed toward non-academic purposes. This study was cross-sectional; thus, causality could not be determined. There may be a possibility of response bias occurrences due to self-reported data collection. Additionally, not everyone in the above average Internet user group in this study should be interpreted as a problematic Internet user. It is necessary to examine the changes in Internet usage time and their influences in the long term. Although this study was a large scale study, data were collected from a homogeneous population of high school adolescents; thus the findings should be generalized with caution.

## Conclusion

We analyzed the relationship between Internet usage time and mental health among adolescents and observed that the above average Internet usage group differed from the below-to-average group in mental health; specifically, in regards to subjective health status, level of stress, feelings of sadness, and suicidal ideation. The results suggest the need for interventions that increase the awareness of the risks for adolescents’ mental health and management of mental health for those adolescents who spend an excessive amount of time using the Internet. Therefore, it is necessary to comprehensively promote the importance of healthy Internet usage. As a means to reduce feelings of sadness, suicidal ideation, and stress among adolescents, educational programs that teach appropriate Internet usage and duration of Internet use need to be developed and implemented.

## References

[1] International Telecommunication Union. Measuring digital development. Facts and figures 2020. Available at: https://www.itu.int/en/ITU-D/Statistics/Documents/facts/FactsFigures2020.pdf. Accessed June 4, 2021.

[2] Griffiths MD. Facebook addiction: concerns, criticism, and recommendations—a response to Andreassen and colleagues. *Psychol Rep*. 2012;110(2):518–520. doi: 10.2466/01.07.18.PR0.110.2.518-520 22662405

[3] Herrero J, Urueña A, Torres A, Hidalgo A. Socially connected but still isolated: Smartphone addiction decreases social support over time. *Soc Sci Comput Rev*. 2019;37(1):73–88.

[4] Frangos CC, Frangos CC, Sotiropoulos I. Problematic internet use among Greek university students: an ordinal logistic regression with risk factors of negative psychological beliefs, pornographic sites, and online games. *Cyberpsychol Behav Soc Netw*. 2011;14(1–2):51–58. doi: 10.1089/cyber.2009.0306 21329443

[5] Herman KM, Hopman WM, Sabiston CM. Physical activity, screen time and self-rated health and mental health in Canadian adolescents. *Prev Med*. 2015;73:112–116. doi: 10.1016/j.ypmed.2015.01.030 25660484

[6] Kim EG. The relationship between internet use and health behaviors among adolescents. *J Korean Acad Community Health Nurs*. 2015;26(1):52–60.

[7] Rikkers W, Lawrence D, Hafekost J, Zubrick SR. Internet use and electronic gaming by children and adolescents with emotional and behavioural problems in Australia–results from the second Child and Adolescent Survey of Mental Health and Wellbeing. *BMC public health*. 2016;16(1):399–414. doi: 10.1186/s12889-016-3058-1 27178325 PMC4866411

[8] Tsitsika AK, Andrie EK, Psaltopoulou T, Tzavara CK, Sergentanis TN, Ntanasis-Stathopoulos I, et al. Association between problematic internet use, socio-demographic variables and obesity among European adolescents. *Eur J Public Health*. 2016;26(4):617–622. doi: 10.1093/eurpub/ckw028 27114408

[9] Romer D, Bagdasarov Z, More E. Older versus newer media and the well-being of United States youth: results from a national longitudinal panel. *J Adolesc Health*. 2013;52(5):613–619. doi: 10.1016/j.jadohealth.2012.11.012 23375827

[10] Shadzi MR, Salehi A, Vardanjani HM. Problematic internet use, mental health, and sleep quality among medical students: A path-analytic model. *Indian J Psychol Med*. 2020;42(2):128–135. doi: 10.4103/IJPSYM.IJPSYM_238_19 32346253 PMC7173655

[11] Kim J-I. The Effects of Sociality, Life Stress, and Depression on the Smartphone Addiction of Nursing College Students. *J Korea Acad-Ind Coop Soc*. 2020;21(4):100–108.

[12] Yun Y-D, Ji H-S, Lim H-S. A study on the analysis of factors that influence Internet usage of adolescence. *J Korean Association Comput Educ*. 2016;19(5):55–71.

[13] Ministry of Gender Equality and Family Statistics Korea. 2018 Youth statistics in Korea. Press release (2019).

[14] Durak HY. Modeling of variables related to problematic internet usage and problematic social media usage in adolescents. *Curr Psychol*. 2018;39:1375–1387.

[15] Günlü A, Ceyhan AA. Investigating adolescents’ behaviors on the internet and problematic internet usage. *Turkish J Addict*. 2017;4(1):96–117.

[16] Kocaman O, Aktepe E, Sonmez Y. An examination of the relationship between possible internet addiction and the levels of aggression and empathy among high school students in Isparta province/Isparta il merkezi lise ogrencilerinde olasi internet bagimliligi ile saldirganlik ve empati duzeyleri arasindaki iliskinin incelenmesi. *Psikiyatri Dergisi*. 2017;18(6):602–611.

[17] Zorbaz O, Tuzgöl Dost M. Examination of Problematic Internet Use of High School Student in Terms of Gender, Social Anxiety and Peer Relations. *HÜEF-Hacettepe Uni J Edu*. 2014;29:298–310.

[18] Ceyhan A, Ceyhan E. The Validity and reliability study of problematic internet usage scale for adolescent. *J Dependence*. 2014;15:56–64.

[19] Puri A, Sharma R. Internet usage, depression, social isolation and loneliness amongst adolescents. *Indian J Health Wellbeing*. 2016;7(10):996–1003.

[20] Goswami V, Singh DR. Impact of mobile phone addiction on adolescent’s life: A literature review. *Int J Home Sci*. 2016;2(1):69–74.

[21] Park C-M, Kim Y-B. Health promotion services and administrative system of the university health clinic. *Korean J Health Educ Promo*. 2010;27(2):151–163.

[22] Aktepe E, Erturan I, Isik A. Evaluation of problematic Internet usage, characteristics of Internet usage, and other related psychiatric factors in adolescents with acne. *Dermatologica Sinica*. 2020;38(1):9–14.

[23] Kuss D, Griffiths M, Karila L, Billieux J. Internet addiction: A systematic review of epidemiological research for the last decade. *Curr pharm des*. 2014;20(25):4026–4052. doi: 10.2174/13816128113199990617 24001297

[24] Moon JH, Song ES, Seeong HY. Investigation of Self-Rated Health and Happiness, Physical Activity, and Mental Health by Smartphone Overuse using Korea Youth Risk Behavior Web-Based Survey 2017. *J Korea Entertain Ind Association*. 2019;13(8):515–524.

[25] Cao H, Sun Y, Wan Y, Hao J, Tao F. Problematic Internet use in Chinese adolescents and its relation to psychosomatic symptoms and life satisfaction. *BMC public health*. 2011;11(1):802–810. doi: 10.1186/1471-2458-11-802 21995654 PMC3214169

[26] Sami H, Danielle L, Lihi D, Elena S. The effect of sleep disturbances and internet addiction on suicidal ideation among adolescents in the presence of depressive symptoms. *Psychiatry res*. 2018;267:327–332. doi: 10.1016/j.psychres.2018.03.067 29957549

[27] Biddle L, Derges J, Mars B, Heron J, Donovan JL, Potokar J, et al. Suicide and the Internet: Changes in the accessibility of suicide-related information between 2007 and 2014. *J Affect Disord*. 2016;190:370–5. doi: 10.1016/j.jad.2015.10.028 26546772

[28] Szumilas M, Kutcher S. Teen suicide information on the internet: a systematic analysis of quality. *Can J Psychiatry*. 2009;54(9):596–604. doi: 10.1177/070674370905400904 19751548

[29] Kim EY, Song MH, Kim YJ. The effect of newspaper suicide reporting on suicide-related perception: focusing on the dynamic relationship between suicide contents and web-search activities. *Korean J Journal Commun Stud*. 2015;59(3):94–122.

[30] Cheng Y-S, Tseng P-T, Lin P-Y, Chen T-Y, Stubbs B, Carvalho AF, et al. Internet addiction and its relationship with suicidal behaviors: a meta-analysis of multinational observational studies. *J Clin Psychiatry*. 2018;79(4):17r11761. doi: 10.4088/JCP.17r11761 29877640

